# Evaluation of the Toxicity Profile and Central Nervous System Activities of Glue (Adhesive) Inhalation in Wistar Rats

**DOI:** 10.1155/jt/5535209

**Published:** 2025-04-28

**Authors:** Abdulgafar Olayiwola Jimoh, Shuaibu Abdullahi Hudu, Onyinye Emmanuella Ibeabuchi, Bilyaminu Abubakar, Millicent Ladi Umaru, Zuwaira Sani, Edith Ginika Otalike, Umar Mohammed, Muhammad Sanusi Haruna, Kehinde Ahmad Adeshina

**Affiliations:** ^1^Department of Pharmacology and Therapeutics, Faculty of Basic Clinical Sciences, College of Health Sciences, Usmanu Danfodiyo University, Sokoto, Nigeria; ^2^Department of Basic and Clinical Medical Sciences, Faculty of Dentistry, Zarqa University, P.O. Box 132222, Zarqa 13132, Jordan; ^3^Department of Medical Microbiology and Parasitology, Faculty of Basic Clinical Sciences, College of Health Sciences, Usmanu Danfodiyo University, Sokoto, Nigeria; ^4^Department of Pharmacology and Toxicology, Faculty of Pharmaceutical Sciences, Usmanu Danfodiyo University, Sokoto, Nigeria; ^5^Department of Family Medicine, Usmanu Danfodiyo University Teaching Hospital, Sokoto, Nigeria; ^6^Department of Morbid Anatomy and Forensic Medicine, Usmanu Danfodiyo University, Sokoto, Nigeria; ^7^Department of Physiology, Faculty of Basic Medical Sciences, Federal University of Health Sciences, P.M.B. 45, Azare, Nigeria; ^8^Department of Physiology, Faculty of Basic Medical Sciences, College of Health Sciences and Centre for Advanced Medical Research and Training (CAMRET), Usmanu Danfodiyo University, Sokoto, Nigeria

**Keywords:** behaviour, central nervous system, depression, glue, toxicity

## Abstract

**Background:** Glue inhalation is a common unconventional substance of abuse, and it contains neurotoxic and volatile solvents. We examined the toxicity profile and central nervous system (CNS) activities of glue inhalation in Wistar rats.

**Methods:** Acute toxicity was investigated, and the subacute toxicity was studied using 24 male Wistar rats at graded concentrations of air, 2, 4 and 8 mL glue (*n* = 6/group) for 28 days. Blood samples were collected for biochemical and haematological evaluations, and vital organs (lung, liver, kidney, heart, brain and eye) were used for histological analysis. Behavioural studies were carried out using an elevated plus maze, hole board test, open-field test, tail suspension test and forced swim test. Dependence and abstinence effects were also evaluated.

**Results:** The lethal dose (LD_50_) of the inhalational glue was 14.14 mL. Glue significantly increased liver function parameters such as TB, DB, ALP, ALT, TP and electrolyte levels (K^+^ and HCO_3_) but reduced cholesterol levels in exposed rats. Glue inhalation had significant anxiolytic and depressant effects on the rats at concentrations of 4 and 8 mL. Histological analysis revealed liver and lung tissue abnormalities and subconjunctival inflammation in eye tissue at 8 mL.

**Conclusion:** The study therefore suggests that glue inhalation has anxiolytic and depressant effects in Wistar rats.

## 1. Introduction

Drug abuse is the excessive use of psychoactive, prescription or over-the-counter drugs, for purposes other than those for which the drug is meant. The abuse of drugs causes a high-risk individual to engage in violent and aggressive behaviour by altering brain activities. There is a complex relationship between drug abuse and violence, with intoxication, neurotoxic and withdrawal symptoms often being mixed up and misinterpreted [1]. Nigeria, being the most populous country in Africa, has evolved to be a centre for the usage and trafficking of drugs, commonly among the population's youth [2]. Due to strict regulations by the United Nations Office on Drugs and Crime (UNODC) and the National Drug Law Enforcement Agency (NDLEA), many adolescents and young adults have turned to unregulated and unconventional substances as alternatives to achieve psychoactive effects. There is an estimated report that 18 in 20 people aged 12 years or older depend on psychoactive substance [3].

These substances can be classified based on their algological composition or based on their effects on the substance users [4]. Psychoactive substances have been identified to be abused in Nigeria and include volatile solvents, plant-based psychoactive substances, lizard dung/excretes, cocoa paste mixed with tobacco, glue, soak away/pit toilet fumes (biogeneric gas) and other psychoactive faunas. Inhalation of volatile substances for their euphoric effects is known as inhalant abuse [5, 6]. Glues and other adhesives have been abused for years, and other solvents such as toluene and petroleum are also sniffed; hence, the term ‘solvent abuse' is given to this dangerous habit, and huffing, bagging, dusting and sniffing are the different ways of inhalation abuse [4].

Glue is a sticky nonmetallic substance used to join two separate objects together by application on both surfaces and allowed to dry, often used for the repair of broken things. The inhalation of this substance is said to be a bedrock of the abuse of more addictive drugs. Glue inhalation has been reported to be a common problem among street children of countries in continents such as Asia, America and Africa [4, 5]. Abusers who seek its euphoric effect are typically teenagers and younger adults who inhale glue to get ‘high' also young adults of low-income population seeking alternative means of getting high from unconventional substances [3].

Glue inhalation is one of the most commonly abused unconventional substances due to its widespread availability and integration into daily activities. Its ease of access and affordability make it a growing public health and socioeconomic concern globally [3]. As a rising health concern in Nigeria with Northern Nigeria taking the lead, the main active constituent in glue is toluene [3, 4]. Toluene exposure may result in chronic respiratory system, cardiac malfunction, euphoria, hallucinations, depression, cognitive dysfunction, ataxia, sedation, mental confusion, unconsciousness, insomnia, headaches, renal malfunction, liver and peripheral nerve damage, hypoxia and hearing loss [6, 7]. Toluene is suspected to be an anxiolytic agent with CNS depressant activity [8, 9]. Sniffing of glue can also lead to abdominal pain in children [4, 10]. Despite the high prevalence of glue inhalation or sniffing and the serious consequences recorded in so many studies, not enough is known about the behavioural effects of abusing glue [1, 6]. The primary solvents contained in rubber solution have been broadly studied individually but not as a mixture (glue), and its withdrawal effects are not well known; hence, the necessity for further studies with animal models properly designed to explore the effect of the solvents and to evaluate the CNS activities and toxicity profile of glue inhalation [4, 11]. We, thus, evaluated the anxiolytic and depressant effects of glue inhalation, including the withdrawal effects of glue inhalation after 2 weeks of abstinence from glue in Wistar rats. A preprint has previously been published [4].

## 2. Materials and Methods

### 2.1. Experimental Animals

Only male Wistar rats were used in this study to minimise potential variability due to hormonal fluctuations that occur in female rats during the oestrous cycle. Seventy-two male Wistar rats weighing between 145-160 g were obtained from the animal house, Faculty of Pharmaceutical Sciences, Amadu Bello University, Zaria. The animals were housed in standard laboratory conditions at the Animal Care Facility of Usmanu Danfodiyo University, Sokoto, Nigeria. The temperature was maintained at 22°C–25°C, with relative humidity of 50%–60%. The animals were kept under a 12-h light/dark cycle. They were provided with standard rodent feed and water ad libitum. The cages were cleaned regularly to maintain hygiene.

### 2.2. Glue (Adhesive)

The inhalant used in this study was the ‘diamond rubber solution' by Anhui Morshine International Cp., Ltd., Hefei, Anhui, China, available in tubes of 15–20 mL. It contains toluene, benzene and other volatile organic solvents [12]. It is used for patching up tyres, and based on verbal communications, it is the most commonly abused type of glue (adhesive) in Sokoto State, Nigeria.

### 2.3. Inhalation Protocol

Each animal was housed in semihermetic plastic chambers (26 × 17 × 15 cm) containing corncob bedding and wire tops. The chambers had aeration holes to ensure consistent airflow throughout the experiment. The glue was put into a small glass dish covered by a grid (to prevent direct contact between the animals and the glue) and set on one side of the inhalation chamber. An empty dish was placed for the control groups on the other end of the inhalation chamber. Immediately after the exposure sessions (4 h for acute toxicity study and 30 min for subchronic study), the rat was removed and returned to their habitual cages [1].

### 2.4. Toxicity Study

#### 2.4.1. Acute Toxicity Study

The LD_50_ of the glue inhalation was determined using the modified Lorke's method [10] and the OECD guideline for inhalation toxicity [13]. This test was carried out in two phases.

  Phase 1: Three groups of three rats each (*n* = 3) were exposed to (0.5, 1 and 2 mL of glue) for 4 h, respectively, after which the rats were immediately removed, returned to their cages and observed for 24 h for signs of acute toxicities and possible mortality.
  Phase 2: Three [3] groups of rats (*n* = 1 for each) were exposed to (5 mL, 10 mL and 20 mL of glue) for 4 h, respectively. Vital organs of the animal with mortality were extracted and taken for histological studies, while the other animals were returned to their habitual cages and observed for 24 h and 14 days, for any sign of toxicity and possible mortality. The LD_50_ was identified by determining the geometric mean of the highest dose survived by the rat and the lowest dose that led to mortality.

(1) $$\begin{array}{c} {\text{LD}}_{50}=\sqrt{D_{0}}\times D_{100}, \end{array}$$

where D_0_ is the highest dose that the animal survived and D_100_ is the lowest dose that caused the death of the animal. The findings from the acute toxicity study were used to determine the duration of exposure and quantity of glue that was used in the subacute and behavioural studies.

#### 2.4.2. Subacute Toxicity Study

Using the guidelines of the Organisation for Economic Cooperation and Development (OECD 412) [13], twenty-four male Wistar rats were randomly distributed using decision analysis, into four groups of six rats each (*n* = 6): control and experimental group, and a dish containing glue was placed in the inhalational chamber of the experimental group, while an empty dish was placed in the centre of the control inhalation chamber. The first group was exposed to air, while the second, third and fourth groups inhaled graded concentrations of (2, 4 and 8 mL) glue for 30 min daily for 28 days, with daily observation of general signs of toxicity and mortality being carried out. The animals were weighed and recorded weekly, and on the 29th day, the animals were euthanised under general anaesthesia, using diethyl ether. Blood samples were collected via cardiac puncture, and whole blood was collected in EDTA bottles for haematological evaluations, and the serum was collected in plain bottles for biochemical analysis, while histopathological examination was performed on the lungs, livers, kidneys, hearts, brains and eyes tissues.

### 2.5. Biochemical Study

The following parameters were determined from the blood serum: albumin, total protein, alkaline phosphatase (ALP), alanine aminotransferase (ALT), aspartate aminotransferase (AST), total bilirubin, direct bilirubin, glucose, serum electrolyte, urea and creatinine, at the Biochemistry Laboratory of Usmanu Danfodiyo University Teaching Hospital, Sokoto State, by using methods in [11, 14] and also using an automatic chemistry analyser and adhering to the manufacturer's manual (Randox).

### 2.6. Histological Study

A qualified histopathologist, unaware of the experimental groups to which each animal belongs to, was employed for all histological examinations of the lungs, livers, kidneys, hearts, brains and eyes tissues.

### 2.7. Slide Preparation

After sacrifice, the organs were extracted, rinsed with tap water and stored in 10% formalin, except for the eye and brain tissues that was stored in burn solution. Each organ tissue was cut transversely and put into separate cells, after which the tissue was processed using tissue processing machine for internal support. It was embedded with paraffin for external support, trimmed and then cut into tiny sections of 3–5 microns and then placed on a frosted glass slade. It was dewaxed and hydrated and then stained with haematoxylin and eosin stain (H and E). It was mounted with dibutyl phthalate polystyrene xylene (DPX) and viewed with an X40 objective lens.

### 2.8. Haematological Study

The following haematological tests were carried out on blood samples, collected from all animals in each group and placed in EDTA bottles: white blood cells (WBC), red blood cell (RBC), granulocyte (GRA), lymphocyte (LYM), haemoglobin (Hb), haematocrit (HCT), mean corpuscular volume (MCV), mean corpuscular Hb (MCH), mean corpuscular Hb concentration (MCHC), red cell distribution width (RDW), platelets (PLT) and mean platelet volume (MPV). Using the method in [11], haematological study was performed using an automatic haematology analyser (AHA) available in Haematology Laboratory of Medistop Diagnostic Centre, Mabera, Sokoto State.

### 2.9. Experimental Design to Measure CNS Activity

The design for this experiment was adopted from Saha and Banerjee [15]. Thirty-six male Wistar rats were randomly distributed using decision analyst into six groups with six animals in each group: Group I: control group received only air; Group II: low concentration of glue inhalation (2 mL); Group III: intermediate concentration of glue inhalation (4 mL); Group IV: high concentration of glue inhalation (8 mL); Group V: diazepam 1 mg/kg p.o or imipramine 10 mg/kg p.o dissolved in vehicle depending on the test. Animals were exposed for at least 3 weeks before the behavioural study, and the test was carried out between 6:00 p.m.−12:00 a.m.

### 2.10. Anxiolytic Activity Using Elevated Plus Maze (EPM)

The plus maze apparatus consisted of two open arms (without walls) (50 × 10 cm) and two enclosed arms (50 × 10 cm) with a high wall of 40 cm arranged in a way that similar arms are directly opposite to each other, with a central square of 10 cm which looks like a plus sign [16]. The apparatus was elevated by a support, 50 cm above floor level. The method was adopted from [15]. Rats from Groups I, II, III, IV and V as stated in the experimental design were included in this study. The Group V received 1 mg/kg of diazepam orally 30 min prior to the test, while Groups I, II, III and IV were exposed to air, 2, 4, and 8 mL of glue, respectively, 30 min prior to the test. Each rat was placed individually at the centre of the EPM with its head facing toward an open arm, and away from the observer. The test lasted for 5 min, and the following parameters were observed during the period with the help of a camera: (a) time spent in open arm, (b) number of entries into open arm and (c) time spent in closed arm.

### 2.11. Anxiolytic Activity of Glue Inhalation Using ‘Hole-Board' Test (HBT)

Rats from Groups I, II, III, IV and V as stated in the experimental design above were included in this study. This method was adopted from [15]. Group V received 1 mg/kg of diazepam orally 30 min prior to the test, while Groups I, II, III and IV were exposed to air, 2, 4 and 8 mL of glue, respectively, 30 min prior to the test. Animals from each group were individually placed in the centre of the hole-board and away from the observer, the test lasted for 5 mins, and the following parameters were observed during the period with the help of a camera: (a) number of head dips, (b) number of rearing, (c) the latency of the first head dip and (d) spontaneous movements (number of squares crossed with all four paws).

### 2.12. Anxiolytic Activity Using Open-Field Test (OFT)

The open-field area was made of plain wood and consists of a square area (72 × 72 × 35 cm). The floor had a square sheet of wood (72 × 72 cm) with the surface divided into 16 squares (18 × 18 cm). One side of the wall was made of plexiglass material for visibility of the animal inside the box. The apparatus was illuminated by a 60 W bulb placed at a height of 100 cm. The method was adopted from [15]. Rats from Groups I, II, III, IV and V as stated in the experimental design above were included in this study. Group V received 1 mg/kg of diazepam orally 30 min prior to the test, while Groups I, II, III and IV were exposed to air, 2, 4 and 8 mL of glue, respectively, 30 min prior to the test. Each rat was placed in the centre square of the apparatus, away from the observer, and the following parameters were recorded: (a) number of lines crossed, (b) number of centre square entries, (c) time spent in centre square, (d) time spent in the periphery, (e) number of rearing, (f) number of grooming and (g) number of assisted rearing [17]. Locomotion was also measured by counting the number of quadrants each animal crossed with all four paws [18].

### 2.13. CNS Depressant Activity Using Forced Swim Test (FST)

We used a vertical transparent cylinder tank (height: 40 cm; diameter: 18 cm, containing water to a height of 15 cm) and maintained at a temperature of 25°C [19]. Rats from Groups I, II, III, IV and V as stated in the experimental design above were included in this study. Group V received 10 mg/kg of imipramine orally, 1 hour prior to the test, while Groups I, II, III and IV were exposed to air, 2, 4 and 8 mL of glue, respectively, 30 min prior to the test. Each rat was placed gently by the tail into the water tank with no possible escape route and forced to swim. The total time spent floating or immobile was recorded with help of a camera. The test was performed under normal illumination, although the rats were allowed to acclimatize with the test environment during the first 60 s off the six minutes test session, leaving only the last five minutes to be analysed. The rats were closely monitored throughout the test period in case of drowning. On completion of the test, the rats were removed from the tank and wiped dry with a towel before placing them back in their home cages. Immobility time was considered a measure of learned helplessness or behavioural despair.

### 2.14. CNS Depressant Activity Using Tail Suspension Test (TST)

Rats from Groups I, II, III, IV and V as stated in the experimental design above were included in this study. Group V received 10 mg/kg of imipramine orally, 1 hour prior to the test, while Groups I, II, III and IV were exposed to air, 2, 4 and 8 mL of glue, respectively, 30 min prior to the test. The rats were suspended over the edge of a shelf, with its nose approximate 20–25 cm from the floor, using a piece of adhesive tape, about 17 cm long, of which 2 cm was used to stick the rat (1 cm from the tip of the tail) and the other 15 cm is used for the suspension. The duration of immobility was recorded for a period of 6 min, and immobility time was recorded during the last 5 min. Immobility can also be recorded for the entire duration of suspension as long as it is consistent with all rats and Groups. The total immobility time was recorded for each rat with the help of a camera. Animal was considered immobile when they hang passively and completely motionless.

### 2.15. Withdrawal Effect Following Glue Inhalation Exposure

After 28 days of exposure to glue inhalation using the same inhalation protocol described in the subacute study above the Wistar rats were abstained from glue exposure.

### 2.16. Conditioned Place Preference (CPP)

Before withdrawal effects could be studied, dependence was tested using a modified ‘CPP' method adopted from [20, 21]. The animals in Groups I, II, III and IV from the experimental design above were subjected to the CPP test, consisting of three distinct compartments separated by two guillotine doors. The walls of the middle chamber were grey with a smooth floor, and while the conditioned compartment (21 × 21 × 27.5 cm, internal volume of 12 L) had black walls with a smooth floor, the unconditioned compartment had white walls with a smooth floor. The lids of the three compartments were made of transparent plexiglass, and the compartmental barriers were guillotine doors. The wire gauze in the brown box was used to introduce glue while the openings on the top of the white and brown compartments were used to introduce air, respectively, under positive pressure. Animals were introduced into the chambers by opening the lid which was immediately closed afterwards and left to move freely for 15 min, and the procedure was monitored with the help of a video. The test was carried out in three phases; the Day 1 was the baseline, the Day 2 was the conditioning (drug) and the Day 3 was the main test. Animals showing preference to a particular box were exempted from the study.

### 2.17. Withdrawal Effect

After the CPP test, the rats were abstained from air and glue inhalation, for 14 days. The rats involved were observed for any physical sign of withdrawal such as anxiety, seizures and behavioural changes that are potentially life-threatening [21]. On the 15th day, the animals were euthanised, and blood samples were collected for biochemical and haematological for any sign of recovery.

### 2.18. Statistical Analysis

Data were stored in Microsoft Excel, while data analysis was performed using Statistical Package for Social Science (SPSS) Version 19. Results were presented in percentages, tables and figures. The results were summarised as mean ± standard error of mean. One-way ANOVA was performed to test the statistical difference with the post hoc test using Tukey multiple comparison test for the normally distributed data. Test analysis was considered significant at *p* < 0.05.

## 3. Results

### 3.1. Inhalational Acute Toxicity Studies of Glue Inhalation

During the acute toxicity test, mortality was observed in Phase II of acute toxicity study, at the highest concentration of 20 mL during the last minutes of a 4-h exposure to glue with a median lethal concentration of 14.14 mL. Additionally, the animals exhibited a series of exploratory behaviour such as rearing, grooming (rubbing of the forelimbs against the nose), seizure, salivation and then followed by sedative effects and significantly abnormal gait.

### 3.2. Effect of Glue Inhalation on Liver Function Test, Following 28 Days Subacute Exposure in Wistar Rats

The result of the liver function analysis showed that there was significant increase (*p* < 0.05) in some of the liver function parameters at various concentrations including total protein, total bilirubin, direct bilirubin, ALP and ALT, and a significant decrease in cholesterol following 28 days of glue inhalation (Table 1).

### 3.3. Effects of Glue Inhalation on Renal Function Test Following 28 Days Subacute Toxicity Study

Result from the renal function analysis showed significant changes (*p* < 0.05) in some of the renal function parameters, with increased urea concentration at 8 mL and increased serum electrolyte level in particular potassium and bicarbonate at 2, 4 and 8 mL, following 28 days of glue inhalation (Table 2).

### 3.4. Effects of Glue Inhalation on Haematological Indices Following 28 Days Subacute Toxicity Study

There was a marked reduction in GRA level and increased platelet and LYM levels although not statistically significant at all concentrations (Table 3).

### 3.5. Histological Analysis of Organs Following 28 Days of Glue Inhalation

**Figure 1:**
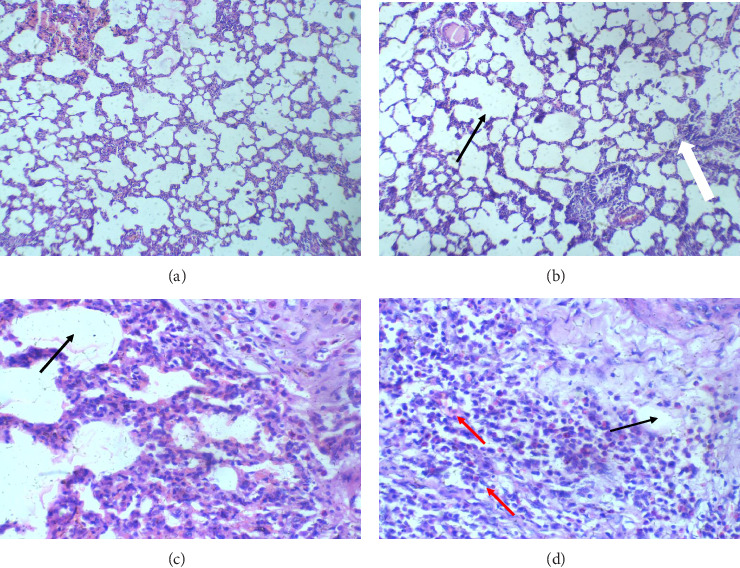
Lung sections H and E X 100 (a–d). (a) (control): normal regular alveoli space and interstitium. (b) (2 mL of glue): normal regular alveoli space (black arrow), mild interstitium inflammatory infiltrate (white arrow) (c) (4 mL of glue) and (d) (8 mL of glue): normal regular alveoli space (black arrow) and with moderate interstitium inflammatory infiltrate (white arrow).

The lungs: In the control group (air exposure), the lungs display a normal histology, with regular alveolar spaces and interstitium. However, in Group II, exposed to 2 mL of glue, the mild interstitial inflammation was observed, though the alveolar spaces remain regular. This mild inflammation intensifies with increased exposure in Group III (4 mL), where moderate interstitial inflammation was observed, while alveolar spaces continue to maintain their structure. Group IV, exposed to the highest volume of glue (8 mL), also shows moderate interstitial inflammation, similar to Group III, suggesting no further structural changes in alveolar spaces. Thus, the lungs demonstrate a dose-dependent inflammatory reaction, progressing from mild-to-moderate interstitial inflammation as glue exposure increases, but alveolar architecture remains intact (Figure 1).

**Figure 2:**
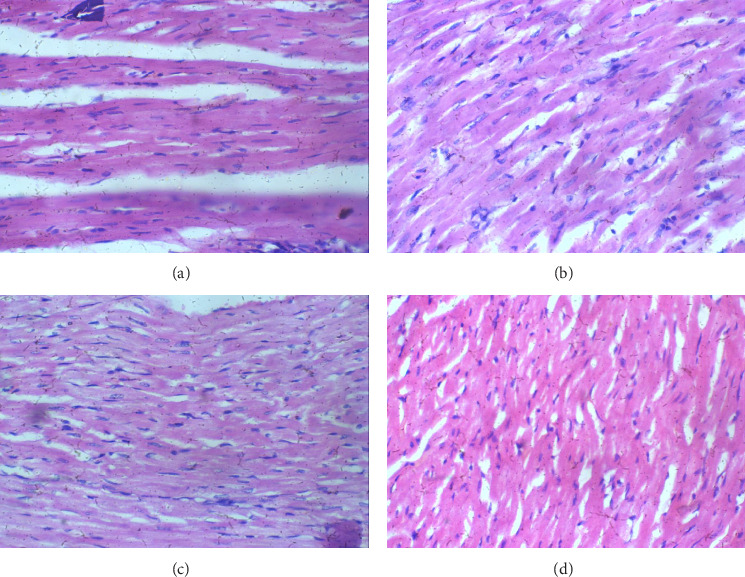
Heart sessions H and E X 100 (a–d). (a) Control, (b) 2 mL of glue, (c) 4 mL of glue, (d) 8 mL of glue. Heart sections show regular cardiac myocytes.

The heart: Tissues appear unaffected across all groups, maintaining regular histological structure with healthy cardiac myocytes in each case, and no visible signs of inflammation or other cellular changes. Therefore, the heart appears resistant to the effects of glue exposure in this study, as cardiac myocytes show no signs of inflammation or structural changes at any exposure level (Figure 2).

**Figure 3:**
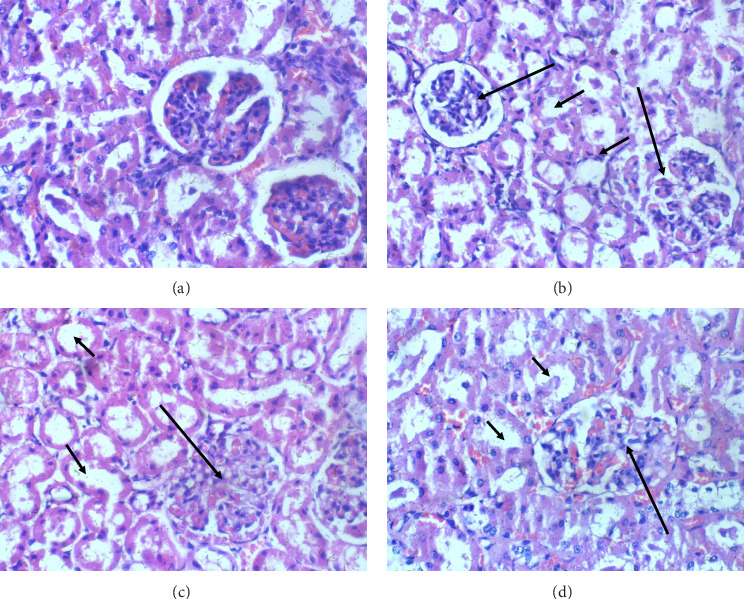
Kidney sections H and E X 100. (a) Control: Kidney section shows regular glomeruli and renal tubules, (b) 2 mL of glue, (c) 4 mL of glue, (d) 8 mL of glue. Kidney sections (b–d) all show regular glomeruli (long arrow) and renal tubules (short arrow).

The kidneys: Across all groups, including the control and varying glue exposure levels, kidney histology consistently appears normal with regular glomeruli, tubules and interstitial structure without any signs of inflammation or cellular changes. This consistency across groups suggests that the kidneys are resistant to any histopathological impact from glue exposure at the levels tested in this study (Figure 3).

**Figure 4:**
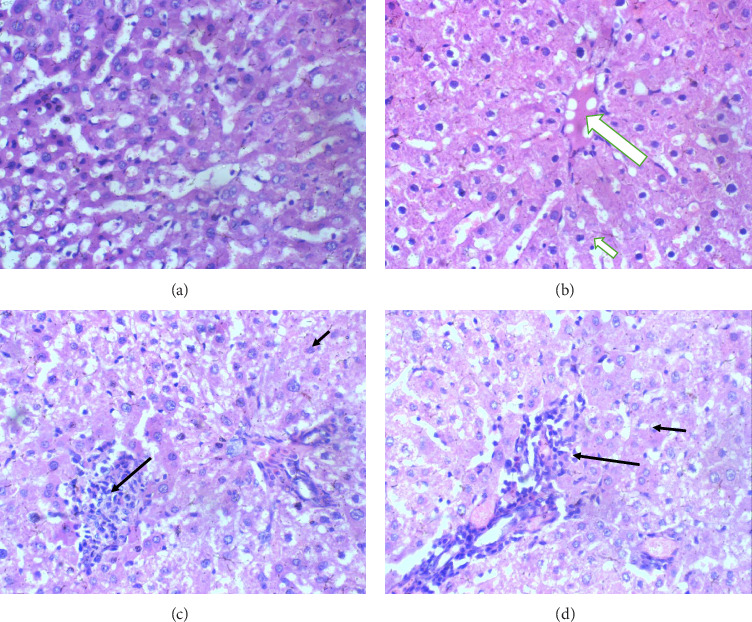
Liver sections H and E X 100. (a) Control and (b) 2 mL of glue: normal central vein (long arrow) and hepatocytes arrange in cords (short arrow). (c) 4 mL of glue: mild porta triaditis (long arrow) and hepatocytes arrange in cords (short arrow). (d) 8 mL of glue: moderate porta triaditis (long arrow) and hepatocytes arrange in cords (short arrow).

The liver: In the control group, liver histology appears normal with a well-defined portal triad, central vein and normal hepatocytes, showing no signs of inflammation or structural disturbance. In Group II (2 mL glue exposure), the liver remains unaffected, with the portal triad, central vein and hepatocytes all appearing normal. In Group III (2 mL glue exposure), the liver shows mild portal triaditis, while the hepatocytes and central vein remain regular. In Group IV (8 mL exposure), the liver shows moderate portal triaditis, indicating an increase in inflammation around the portal area. However, the hepatocytes and central vein remain unaffected. Thus, increasing glue exposure results in progressive portal inflammation, ranging from mild to moderate, while the core structures such as hepatocytes and the central vein are preserved (Figure 4).

**Figure 5:**
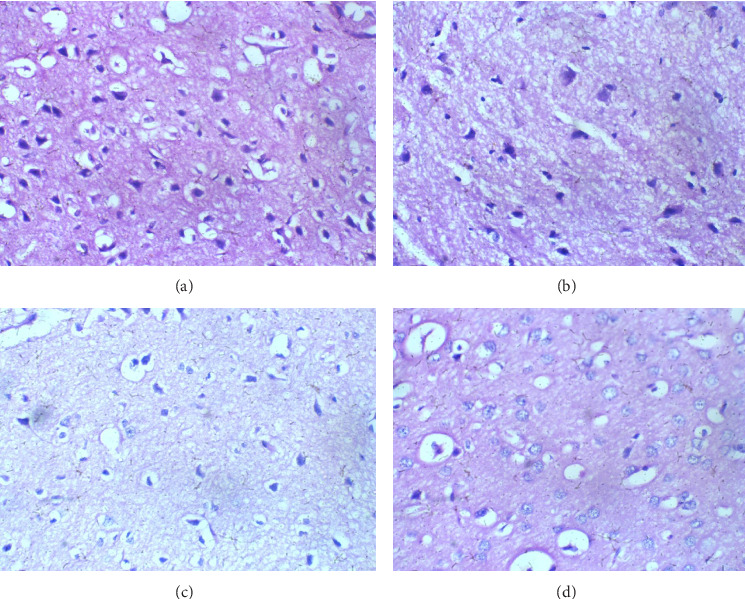
Brain sessions H and E 100. (a) Control, (b) 2 mL of glue, (c) 4 mL of glue, (d) 8 mL of glue: A brain sections show regular neuropils.

The brain: Tissue across all experimental groups, including the control, shows no histopathological differences, maintaining healthy neuropils throughout, with no signs of inflammation or tissue disruption. These findings indicate that the brain is not impacted by glue exposure under the conditions of this study (Figure 5).

### 3.6. Effect of Glue Inhalation on the Behaviour of Male Wistar Rats Using an EPM

Inhalational administration of glue at the dose of 8 mL showed a significant (*p* < 0.05) increase in the duration time spent in the open arms (Table 4) compared to the control group (air), and even so when compared to diazepam at a dose of 1 mg/kg a standard anxiolytic agent. Although not statistically significant, it also decreased the number of entries into the closed arm (Table 5) at concentrations of 2 and 8 mL group compared to the control group, but not as much as the diazepam at a dose of 1 mg/kg.

### 3.7. Effect of Glue Inhalation on the Exploratory Capability of Wistar Rats Using HBT

The result of HBT conducted to evaluate the anxiolytic-like effect of glue inhalation on Wistar rats, showed significant increase (*p* < 0.05) in number of head dips on HBT compared to control group (air) (glue 4 mL vs. control [air]), and more than the standard anxiolytic (diazepam) at a dose of 1 mg/kg (Table 6).

### 3.8. Effect of Glue Inhalation on the Behaviour of Wistar Rats Using OFT

The inhalational administration of glue at a dose of 8 mL showed significant increase (*p* < 0.05) in time spent in centre square, a significant decreased time spent in the periphery, also a significant increase in frequency of entry into centre square and the number of lines crossed in OFT compared to control group, even more than the standard anxiolytic (diazepam) at a dose of 1 mg/kg (Table 7).

### 3.9. Effect of Glue Inhalation on the Duration of Immobility in FST in Wistar Rats

The inhalational administration of glue at a dose of 4 mL showed significant (*p* < 0.05) increase in the duration of immobility in FST compared to the standard antidepressant, imipramine 10 mg/kg and relatively higher than the control (air). Significantly (*p* < 0.05), at dose glue of 8 mL, there was a decrease in the duration of immobility in FST compared to the control (air), more so compared to the standard antidepressant (imipramine) at a dose of 10 mg/kg (Table 8).

### 3.10. Effect of Glue Inhalation on Dependence of Wistar Rats to Glue Inhalation Using CPP Test

The inhalational administration of glue at all concentrations showed no significant (*p* < 0.05) difference between the baseline and the main test of the treated groups compared to the control group (air) following 28 days of glue inhalation, essentially inhalation of glue did not show any dependence property on Wistar rats following 28 days subchronic exposure (Table 9).

### 3.11. Effect of Two Weeks Withdrawal From Glue Inhalation on Liver Function Following 28 Days of Exposure to Glue

There were significant increases (*p* < 0.05) in some of the liver function parameters (total and direct bilirubin, total protein, ALT, ALP, AST and albumin) following a 2-week abstinence from 28 days of glue inhalation at concentrations of 2, 4 and 8 mL in Wistar rats (Table 10).

### 3.12. Effect of 2 Weeks Withdrawal From Glue Inhalation on Renal Function Parameter of Wistar Rats Following 28 Days of Exposure to Glue

There were significant increases in some of the haematological parameters (creatinine, sodium, potassium and bicarbonate) especially at concentrations of 2 and 4 mL, and a significant decrease in the potassium level at 8 mL compared to the control group, following a 2-week abstinence from 28 days of glue inhalation (Table 11).

### 3.13. Effect of 2 Weeks of Withdrawal From Glue Inhalation on Haematological Parameters of Wistar Rats Following 28 Days of Exposure to Glue

There were significant increases (*p* < 0.05) in HCT, platelet count and GRA count, especially at 8 mL of glue, why there was a and a significant decrease in LYM count of the haematological parameters of the Wistar rats following a 2-week abstinence from 28 days of glue inhalation at concentrations of 2, 4 and 8 mL compared to the control group (Table 12).

## 4. Discussion

This study evaluated the effect of abusing a volatile substance exclusively in male Wistar rats. The choice for male Wistar rats was to ensure consistency and reduce variability associated with hormonal influences observed in female rodents. However, it is important to acknowledge that sex-based differences may exist in response to glue inhalation. Cruz and Bowen highlighted that females may exhibit heightened sensitivity to certain volatile solvents due to differences in metabolism, neurochemical activity and hormonal interactions [8]. Future research should explore these potential sex differences to provide a more comprehensive understanding of glue inhalation effects across both sexes.

The exploratory behaviour and sedation observed during the acute toxicity study are such as the findings of Bouchatta et al. (1). In addition, the animal showed signs of recovery between 20 min after exposure; this implied that acute glue sniffing has a short duration of action. The histological analysis of the animal with mortality showed diffuse alveolar wall damage with moderate interstitial inflammation of the lung, acute tubular necrosis in the kidney and a regular hepatocyte with moderate portal triaditis of the liver.

The most widely accepted and validated animal models were used in this study for both anxiety and depression; they were models used to evaluate the anxiolytic properties of diazepam and the antidepressant properties of antidepressants [22, 23]. When a rodent is placed in a new environment, there is a conflict between initially hiding from an unknown risk and the tendency to explore the new environment. Rodents naturally avoid open, bright and elevated spaces; hence, the movement of rats in the EPM is because of two main motivations: the exploratory drive and fear drive which are evoked by new stimuli, so assessing anxiety in rodents is performed by using the ratio of time spent in the open arms to the time spent in the enclosed arm.

The CNS effect of glue inhalation has demonstrated that glue inhalation exhibits significant anxiolytic activity, which is believed to be concentration dependent, the reason the glue (8 mL) group did not show increased immobility time compared with the control group (air) is unknown; however, the glue (2 mL) group showed reduced immobility time compared to the control and may be presented as an antidepressant effect at this dose. The EMP is sensitive to both anxiolytic and anxiogenic agents, and staying in the open arm caused physiological stress which can manifest as increased defecation and/or urination, but exposure to typical anxiolytics such as diazepam or substances with anxiolytic properties increases exploration of these open arms [24]. Therefore, in the EPM, an increase in the time spent in the open arm as against the time spent in the closed arm by the glue is an indication of anxiolytic activity, although glue did not increase the number of entries into the open space. Similarly, in HBT which follows similar principles of fear drive and exploratory drive, the anxiety state of the rat is presumed to be inversely proportional to the number of head dips [24]. The increase in the number of head dips by rats exposed to glue inhalation has suggested an index of anxiolytic activity.

The OFT shows a higher number of lines crossed, which indicates an increase in locomotive activity and/or a lower level of anxiety; more so, increased entry and time spent in the centre square expresses decreased anxiety altered by the anxiolytic activity of the substance; therefore, in the OFT, the increase in number of lines crossed, frequency of entries into centre square and time spent in centre square suggested an index of locomotive activity and anxiolytic activity, with increased in the glue treated group compared to the control group and even the diazepam (positive control). The increased locomotive activity is contrary to the findings of a study that discovered a decrease in motor activity at a high concentration [20]. The FST and TST are models that are based on behavioural despair or helplessness; rodents initially try to escape or save themselves in both tests but eventually exhibit despair (immobility). The observed decrease in the duration of immobility for glue 2 mL groups compared to the control group may suggest an antidepressant activity at this concentration. This study showed an increase in the duration of immobility of the imipramine group contrarily to other studies, this finding may be a result of the differences in the strain of rats or delayed onset of action of imipramine [25].

The active constituents responsible for the anxiolytic and CNS depressant effects of glue are likely toluene and benzene, which have been reported to modulate neurotransmitter activity, particularly by affecting GABAergic, dopaminergic and glutamatergic systems [8]. The glue used in this study, diamond rubber solution, is widely available in Nigeria and frequently abused, particularly among adolescents and street children seeking Euphoria. Toluene has been reported to inhibit NMDA receptor activity while enhancing GABA-A receptor function, leading to its anxiolytic and depressant effects [8, 9]. The CNS action of benzene is not entirely clear, but some studies have proposed that benzene has a CNS depressant activity. Thus, the consumption of volatile substances extensively and excessively is quite dangerous to the users [20].

During the 28-day subacute sessions, the observed concentration-dependent effects are classical signs of CNS activity that can result in CNS functional damage. Continued seizure, for instance, can result in impairment or paralysis of the affected region, abnormal respiration and respiratory depression which are the major causes of mortality in inhalation abuse and can result in the death of a glue sniffer. However, we also observed that the unconscious animals recovered between 5–20 min of discontinued exposure, which may explain why glue sniffers need to keep inhaling the glue to maintain the state of Euphoria which makes it even more dangerous.

At the end of the 28-day subacute test, glue inhalation changed some haematological parameters indicative of infection; there was no decrease in WBC, RBC or HB contrary to the finding by Taiwo and Leite [20], who discovered a decrease in WBC, RBC and HB at doses 320 and 640 mg/L, following exposure to ‘di' glue for 28 days [20]. Similarly, the liver function parameters present findings consistent with [20], who used ‘dia' glue on Wistar rats at 320 and 640 mg/L for 28 days [20]. Inhalation of glue caused elevated serum potassium, bicarbonate and urea concentrations in treated animals which can alter the kidney function and may result in kidney disease or failure. The major organ involved in the regulation of electrolyte levels is the kidney, thereby maintaining homoeostasis, which makes urea and serum electrolytes the commonly requested biochemical tests for renal function assessment [26]. The stable histological findings may be a result of the time limit, a significant damage to the organs may occur following chronic abuse of glue.

This study showed that glue inhalation did not cause addiction in Wistar rats, after 28 days of exposure to glue. However, we observed the effect of withdrawal after subacute abuse of glue inhalation, and the result showed more intense hostility among rats in group glue 4 and 8 mL, 1 week after cessation of glue exposure, although the aggressive behaviour subsided by the end of the second week of abstinence. This finding is in tandem with the result of Bouchatta et al. (1). More so, hostility has been associated with benzodiazepine withdrawal in both humans and animals [27], and previous studies have shown that toluene has GABA_A_ agonistic modulatory effects [8]. It is otherwise safe to say that the behavioural control can be likened to the activation of GABAergic neurons which in turn, synapse with other neurons in control of aggressive behaviours [1, 28].

## 5. Conclusion

The findings from this study suggest that the glue inhalation has significant anxiolytic and CNS depressant effects, which may explain its widespread abuse among young adults seeking a calming or euphoric state. However, prolonged exposure to glue inhalation was associated with hepatotoxicity, renal dysfunction and alterations in haematological parameters, indicating potential risks for long-term organ damage. Chronic inhalant abuse has been linked to neurological deficits, cognitive impairment and psychiatric disorders, which could contribute to the development of substance use disorders and dependence.

While our study did not observe physical dependence in Wistar rats following subacute exposure, the withdrawal-related aggression observed suggests that chronic use could lead to behavioural disturbances similar to those seen in benzodiazepine or alcohol withdrawal syndromes. These findings highlight the need for targeted interventions in addiction treatment, particularly for vulnerable populations such as adolescents and individuals with limited access to conventional psychoactive substances.

Further studies should focus on the molecular targets and neurotransmitter alterations following glue inhalation, particularly changes in dopamine, serotonin and GABA levels in key brain regions such as the prefrontal cortex, hippocampus and striatum. Techniques such as high-performance liquid chromatography (HPLC), immunohistochemistry and western blot analysis could be employed to measure neurotransmitter levels and receptor expression following acute and chronic exposure. Understanding these mechanisms could provide insights into the long-term neuropsychiatric consequences of inhalant abuse and potential pharmacological interventions for managing dependence and withdrawal symptoms.

Given the accessibility and affordability of glue, regulatory measures should be reinforced to restrict sales, particularly to minors and to introduce warning labels about potential neurotoxicity and organ damage. Public health campaigns should focus on raising awareness about the dangers of inhalant abuse, while healthcare professionals should be trained to recognise and manage glue-related toxicity and withdrawal symptoms. Further research is needed to explore potential therapeutic interventions for inhalant addiction including behavioural therapies and pharmacological strategies that target neurotransmitter systems affected by volatile solvents.

## Figures and Tables

**Table 1:** Effect of glue inhalation on liver function indices of Wistar rat.

Parameters	Control (air)	Glue (2 mL)	Glue (4 mL)	Glue (8 mL)
Total bilirubin (mg/dL)	3.15 ± 0.23	3.61 ± 0.16	5.25 ± 0.51^ab^	6.62 ± 0.39^ab^
Direct bilirubin (mg/dL)	1.64 ± 0.14	1.55 ± 0.08^d^	2.02 ± 0.34^d^	3.00 ± 0.14^a^
ALP (μ/L)	42.63 ± 2.49	71.79 ± 6.35^a^	102.49 ± 6.06^ab^	115.07 ± 4.66^ab^
AST (μ/L)	63.51 ± 8.57	72.92 ± 18.54	81.65 ± 17.32	80.13 ± 19.50
ALT (μ/L)	22.48 ± 0.68	72.20 ± 8.61^ac^	96.60 ± 2.19^ad^	67.63 ± 0.64^ac^
Total protein (g/dL)	6.00 ± 0.13	6.45 ± 0.22	6.95 ± 0.78	9.07 ± 0.86^ab^
Cholesterol (mg/dL)	216.64 ± 8.99	136.22 ± 12.47^ac^	198.97 ± 10.35	153.46 ± 10.40^ac^
Glucose (mmol/L)	6.36 ± 0.25	5.96 ± 0.34	5.56 ± 0.30	6.72 ± 0.55
Albumin (g/L)	3.56 ± 0.17	3.56 ± 0.17	3.94 ± 0.11	3.65 ± 0.18

*Note:* Data were expressed as mean ± SEM, *n* = 6. Tukey multiple comparison test.

Abbreviations: ALP = alkaline phosphatase, ALT = alanine transaminase, AST = aspartate transaminase, SEM = standard error of mean.

^a^Means significantly different from control group 1 (air) at *p* < 0.05.

^b^Means significantly different from Group 2 (2 mL) at *p* < 0.05.

^c^Means significantly different from Group 3 (4 mL) at *p* < 0.05.

^d^Means significantly different from Group 4 (8 mL) at *p* < 0.05.

**Table 2:** Effect of glue inhalation on renal function parameters in Wistar rats.

Parameter	Control (air)	Glue (2 mL)	Glue (4 mL)	Glue (8 mL)
Urea (mmol/L)	7.36 ± 0.58	9.06 ± 0.58	9.06 ± 0.55	10.20 ± 0.82^a^
Creatinine (μmol/L)	198.54 ± 20.60	218.82 ± 18.59	145.99 ± 10.88^b^	172.38 ± 12.23
Na^+^ (mmol/L)	141.08 ± 1.37	145.05 ± 1.99	145.67 ± 2.18	145.55 ± 2.58
K^+^ (mmol/L)	4.63 ± 0.14	4.55 ± 0.13^a^	4.73 ± 0.18^a^	5.00 ± 0.10^a^
HCO_3_ (mmol/L)	17.53 ± 0.28	20.50 ± 0.41^a^	19.67 ± 0.42^a^	21.10 ± 0.52^a^

*Note:* Data were expressed as mean ± SEM, *n* = 6, Na^+^ = sodium ion, K^+^ = potassium ion, HCO_3_ = bicarbonate. Tukey multiple comparison test.

Abbreviation: SEM = standard error of mean.

^a^Means significantly different from control group 1 (air) at *p* < 0.05.

^b^Means significantly different from Group 2 (2 mL) at *p* < 0.05.

**Table 3:** Effect of glue inhalation on haematological indices following 28 days subacute study.

Parameters	Control (air)	Glue (2 mL)	Glue (4 mL)	Glue (8 mL)
White blood cell (10^9^/L)	21.65 ± 1.49	21.96 ± 1.43	22.60 ± 1.77	22.26 ± 1.74
Red blood cell (10^12^/L)	6.79 ± 0.65	7.28 ± 0.23	7.03 ± 0.22	7.34 ± 0.24
Haemoglobin (g/dL)	14.58 ± 0.35	15.20 ± 0.36	14.67 ± 0.46	15.00 ± 0.37
Haematocrit (%)	37.28 ± 1.10	37.87 ± 1.05	36.42 ± 1.23	37.62 ± 1.44
Platelet (10^9^/L)	625.33 ± 33.89	614.00 ± 24.03	663.33 ± 44.17	699.00 ± 43.42
Granulocyte (%)	32.87 ± 3.82	24.85 ± 1.53	25.58 ± 2.20	23.35 ± 1.88
Lymphocyte (%)	67.78 ± 1.67	70.23 ± 0.86	71.80 ± 3.61	70.25 ± 1.44

*Note:* Data were expressed as mean ± SEM, *n* = 6, *p* < 0.05.

Abbreviation: SEM = standard error of mean.

**Table 4:** Effect of glue inhalation on the behaviour in Wistar rats using elevated plus maze (open arm).

Groups	Number of entry	Time spent (sec)
Control (air)	0.17 ± 0.17	12.0 ± 5.71
Glue (2 mL)	0.33 ± 0.21	10.0 ± 7.28
Glue (4 mL)	0.17 ± 0.17	43.33 ± 11.46
Glue (8 mL)	0.33 ± 0.21	143.17 ± 46.63^ab^
Diazepam (1 mg/kg)	0.33 ± 0.33	128.50 ± 50.93

*Note:* The results are expressed as mean ± SEM, *n* = 6, *p* < 0.05. Tukey multiple comparison test.

Abbreviation: SEM = standard error of mean.

^a^Means significantly different from control group 1 (air) at *p* < 0.05.

^b^Means significantly different from Group 4 (2 mL) at *p* < 0.05.

**Table 5:** Effect of glue inhalation on elevated plus maze (close arm).

Groups	Number of entry	Time spent
Control (air)	2.67 ± 1.05	205.83 ± 47.97
Glue (2 mL)	1.17 ± 0.17	239.83 ± 26.60
Glue (4 mL)	3.67 ± 0.71	249.67 ± 9.67
Glue (8 mL)	1.67 ± 0.61	107.67 ± 45.21
Diazepam (1 mg/kg)	1.0 ± 0.37	95.17 ± 51.86

*Note:* The results are expressed as mean ± SEM, *n* = 6, *p* > 0.05.

Abbreviation: SEM = standard error of mean.

**Table 6:** Effect of glue inhalation on the behaviour in Wistar rats using hole board test.

Groups	Number of head dips
Control (air)	8.0 ± 2.57
Glue (2 mL)	12.0 ± 3.11^c^
Glue (4 mL)	25.5 ± 3.27^a^
Glue (8 mL)	12.3 ± 2.64^c^
Diazepam (1 mg/kg)	9.0 ± 3.82^c^

*Note:* The results are expressed as mean ± SEM, *n* = 6. Tukey multiple comparison test.

Abbreviation: SEM = standard error of mean.

^a^Means significantly different from control group 1 (air) at *p* < 0.05.

^c^Means significantly different from Group 5 (4 mL) at *p* < 0.05.

**Table 7:** Effect of glue inhalation on the behaviour in Wistar rats using open field test.

Group	TSICS (sec)	TSIPS (sec)	FECS	NLC
Control (air)	0.33 ± 0.33	297.50 ± 5.24	0.17 ± 0.17	24.00 ± 7.06
Glue (2 mL)	0.00 ± 0.00^d^	295.33 ± 0.95^d^	0.00 ± 0.00^d^	42.67 ± 19.84
Glue (4 mL)	4.67 ± 4.27^d^	288.00 ± 6.63^d^	1.00 ± 0.52^d^	69.17 ± 13.25
Glue (8 mL)	14.17 ± 4.43^a^	260.17 ± 11.41^a^	4.00 ± 0.63^a^	75.67 ± 7.22^a^
Diazepam (1 mg/kg)	0.00 ± 0.00^d^	290.83 ± 4.53^d^	0.00 ± 0.00^d^	23.17 ± 3.69^d^

*Note:* The results are expressed as mean ± SEM, *n* = 6, *p* > 0.05. Tukey multiple comparison test.

Abbreviations: FECS, frequency of entry into centre square; NLC, number of lines crossed; SEM, standard error of mean; TSICS, time spent in centre square; TSIPS, time spent in peripheral square.

^a^Means significantly different from control group 1 (air) at *p* < 0.05.

^d^Means significantly different from Group 6 (8 mL) at *p* < 0.05.

**Table 8:** Effect of glue inhalation on the duration of immobility in Wistar rats using forced swim test (FST).

Groups	Duration of immobility (sec)
Control (air)	135.83 ± 14.24
Glue (2 mL)	87.17 ± 26.21^c^
Glue (4 mL)	173.33 ± 9.77^f^
Glue (8 mL)	32.50 ± 14.51^ac^
Imipramine (10 mg/kg)	45.50 ± 9.11^a^

*Note:* The results are expressed as mean ± SEM, *n* = 6, *p* < 0.05. Tukey multiple comparison test.

Abbreviation: SEM = standard error of mean.

^a^Means significantly different from control group 1 (air) at *p* < 0.05.

^c^Means significantly different from Group 5 (4 mL) at *p* < 0.05.

^f^Means significantly different from Group 3 (imipramine 10 mg/kg) at *p* < 0.05.

**Table 9:** Effect of glue inhalation on dependence.

Groups	Base line		Main test	
	TSDC (sec)	TSWC (sec)	TSDC (sec)	TSWC (sec)
Control (air)	507.50 ± 118.00	331.83 ± 110.62	507.50 ± 118.00	331.83 ± 110.62
Glue (2 mL)	721.33 ± 75.23	152.00 ± 65.23	650.33 ± 150.09	237.17 ± 150.59
Glue (4 mL)	663.17 ± 36.52	164.67 ± 20.75	719.33 ± 25.22	140.17 ± 22.95
Glue (8 mL)	583.50 ± 125.80	156.50 ± 113.96	612.17 ± 130.81	227.50 ± 129.67

*Note:* The results are expressed as mean ± SEM, *n* = 6.

Abbreviations: SEM, standard error of mean; TSDC, time spent in dark chamber; TSWC, time spent in white chamber.

**Table 10:** Effect of 2 weeks withdrawal from glue inhalation on liver function indices.

Parameters	Control (air)	Glue (2 mL)	Glue (4 mL)	Glue (8 mL)
Total bilirubin (mg/dL)	0.55 ± 0.01	1.34 ± 0.07^acd^	0.58 ± 0.01^d^	2.52 ± 0.11^a^
Direct bilirubin (mg/dL)	0.31 ± 0.02	0.76 ± 0.01^acd^	0.42 ± 0.01^ad^	1.54 ± 0.01^d^
ALP (μ/L)	55.66 ± 1.81	74.06 ± 1.32^ad^	66.24 ± 1.89^ad^	86.02 ± 3.14^a^
AST (μ/L)	32.02 ± 1.56	46.67 ± 0.16^ad^	48.33 ± 0.07^ad^	65.45 ± 0.09^a^
ALT (μ/L)	43.13 ± 0.33	68.65 ± 0.41^acd^	75.25 ± 0.07^ad^	26.35 ± 0.11^a^
Total protein (g/dL)	6.64 ± 0.03	7.08 ± 0.01^acd^	5.59 ± 0.11^ad^	7.76 ± 0.02^a^
Albumin (g/L)	1.18 ± 0.00	2.11 ± 0.01^acd^	1.66 ± 0.01^ad^	1.38 ± 0.00^a^

*Note:* Data were expressed as mean ± SEM, *n* = 6. Data with superscript represent significant difference at *p* < 0.05. Tukey multiple comparison test.

Abbreviations: ALP = alkaline phosphatase, ALT = alanine transaminase, AST = aspartate transaminase, SEM = standard error of mean.

^a^Means significantly different from control group 1 (air) at *p* < 0.05.

^c^Means significantly different from Group 5 (4 mL) at *p* < 0.05.

^d^Means significantly different from Group 6 (8 mL) at *p* < 0.05.

**Table 11:** Effect of 2 weeks withdrawal from glue inhalation on renal function parameter.

Parameter	Control (air)	Glue (2 mL)	Glue (4 mL)	Glue (8 mL)
Urea (mmol/L)	17.77 ± 0.09	21.05 ± 1.01	24.62 ± 3.90	18.73 ± 0.06
Creatinine (μmol/L)	110.27 ± 0.31	167.34 ± 0.54^ad^	181.71 ± 9.78^ad^	136.41 ± 0.31^a^
Na^+^ (mmol/L)	132.00 ± 1.34	138.67 ± 1.30^ad^	140.83 ± 0.48^ad^	133.33 ± 1.28
K^+^ (mmol/L)	3.80 ± 0.07	4.33 ± 0.10^ad^	4.60 ± 0.08^a^	3.32 ± 0.10^ac^
HCO_3_ (mmol/L)	17.53 ± 0.28	23.22 ± 0.15^ad^	23.25 ± 0.29^ad^	20.75 ± 0.23

*Note:* Data were expressed as mean ± SEM, *n* = 6, Na^+^ = sodium ion, K^+^ = potassium ion, HCO_3_ = bicarbonate. Tukey multiple comparison test.

Abbreviation: SEM = standard error of mean.

^a^Means significantly different from control group 1 (air) at *p* < 0.05.

^b^Means significantly different from Group 4 (2 mL) at *p* < 0.05.

^c^Means significantly different from Group 5 (4 mL) at *p* < 0.05.

^d^Means significantly different from Group 6 (8 mL) at *p* < 0.05.

**Table 12:** Effect of 2 weeks withdrawal from glue inhalation on haematological parameters.

Parameters	Control (air)	Glue (2 mL)	Glue (4 mL)	Glue (8 mL)
White blood cell (10^9^/L)	17.13 ± 0.57	12.14 ± 1.02^a^	18.73 ± 1.94^b^	14.43 ± 1.14
Red blood cell (10^12^/L)	7.69 ± 0.25	7.92 ± 0.29	7.40 ± 0.17	7.86 ± 0.11
Haemoglobin (g/dL)	14.83 ± 0.28	15.27 ± 0.43	14.45 ± 0.27	15.05 ± 0.23
Haematocrit (%)	42.55 ± 0.48	45.67 ± 0.99^a^	41.22 ± 0.52^b^	42.05 ± 0.83^b^
Platelet (10^9^/L)	494.67 ± 12.40	691.83 ± 22.97	598.83 ± 114.50	813.67 ± 41.17^a^
Granulocyte (%)	22.18 ± 0.98	28.93 ± 2.21	24.37 ± 0.97	32.95 ± 4.56^a^
Lymphocyte (%)	74.73 ± 1.34	68.82 ± 2.48	66.57 ± 2.10	62.18 ± 2.62

*Note:* Tukey multiple comparison test.

^a^Means significantly different from the control group 1 (air) at *p* < 0.05.

^b^Means significantly different from Group 4 (2 mL) at *p* < 0.05.

^c^Means significantly different from Group 5 (4 mL) at *p* < 0.05.

^d^Means significantly different from Group 6 (8 mL) at *p* < 0.05.

## Data Availability

The data that support the findings of this study are available from the corresponding author upon reasonable request.
